# Effect of Surface Aluminizing on the Zinc Corrosion Resistance of Fe-20Cr-5B-3Al Alloy

**DOI:** 10.3390/ma18112493

**Published:** 2025-05-26

**Authors:** Shanlong Zhu, Ya Liu, Changjun Wu, Xiangying Zhu, Xuping Su

**Affiliations:** 1 School of Materials Science and Engineering, Changzhou University, Changzhou 213164, China; 17312275507@163.com (S.Z.);; 2 Jiangsu Key Laboratory of Materials Surface Science and Technology, Changzhou University, Changzhou 213164, China

**Keywords:** aluminizing treatment, zinc corrosion resistance, Fe-20Cr-5B-3Al alloy

## Abstract

This study systematically investigates the effect of surface aluminizing treatment on the microstructure of Fe-20Cr-5B-3Al alloy and its corrosion behavior in liquid zinc. Aluminizing treatment was performed using the powder pack method with NH_4_Cl (5 wt.%) as a catalyst. Aluminide layers were prepared on the surface of the Fe-20Cr-5B-3Al alloy, and the microstructure of the aluminide layer was observed and analyzed. The corrosion performance of the alloy in liquid zinc was compared before and after aluminizing treatment in a 128 h corrosion test. The results show that after aluminizing treatment, the α-Fe phase on the alloy surface transforms into the Fe_2_Al_5_ phase, while the original Fe_2_B phase breaks into finer structures that disperse over the Fe_2_Al_5_ phase. The Cr_2_B phase is not affected and maintains its structural integrity. After 128 h of exposure to liquid zinc, although the alloy exhibited good corrosion resistance in liquid zinc, the α-Fe phase was still preferentially corroded, with zinc liquid infiltrating along the α-Fe phase toward the interior. In contrast, after aluminizing treatment, the transformation of α-Fe into Fe_2_Al_5_, which has excellent corrosion resistance, significantly reduces the corrosion rate and enhances the alloy’s resistance to liquid zinc corrosion.

## 1. Introduction

The hot-dip galvanizing process is one of the most widely used methods for effectively preventing the corrosion of steel materials [1]. This process typically involves pre-treating the steel material through cleaning and activation, followed by immersion in molten zinc at an elevated temperature [2]. Through the iron–zinc reaction, a strongly adherent zinc alloy coating forms on the steel surface [3]. However, during the hot-dip galvanizing process, critical components inside the zinc pot are continuously exposed to high-temperature molten zinc. Due to the extremely high temperature and strong chemical reactivity of liquid zinc, the materials used for these components inevitably suffer from corrosion [4]. Metal components that remain exposed to liquid zinc for extended periods often fail to meet the required corrosion resistance, leading to frequent occurrences of corrosion and damage during production [5]. As the corrosiveness of molten zinc intensifies, equipment and components inside the zinc pot undergo severe degradation, which not only affects production efficiency but also increases the frequency of maintenance and replacement, raising operational costs for enterprises [6].

To address the corrosion issues of continuous hot-dip galvanizing production line equipment in high-temperature liquid zinc environments, further research and improvements in materials and processes are necessary to enhance the corrosion resistance and service life of equipment components. Current research on zinc corrosion-resistant materials primarily focuses on two directions: surface treatment and bulk corrosion-resistant materials [7,8,9]. Among bulk corrosion-resistant materials, Fe-B alloys have attracted widespread attention due to their excellent resistance to molten zinc corrosion. In this context, Ma et al. conducted a detailed study on the interfacial and corrosion behavior of oriented Fe_2_B alloys in molten zinc, emphasizing the effects of orientation and lamellar spacing. They revealed that when Fe_2_B is oriented perpendicularly to the corrosion interface, it promotes the formation of a multilayer structure via interface pinning, which greatly improves the alloy’s corrosion resistance in liquid zinc [10,11]. Similarly, Shah et al. explored how silicon content and exposure time influence the interface morphology and cavitation erosion of directionally solidified Fe-B alloys, finding that an appropriate Si addition refines the lamellar spacing of Fe_2_B and optimizes the microstructure, thereby enhancing resistance to cavitation erosion in flowing liquid zinc [12]. Additionally, Liu et al. analyzed the corrosion behavior of Fe-B alloys in molten zinc and the role of silicon. They found that Fe-B alloys corrode at a relatively low and stable rate in flowing liquid zinc and that the Si element in the α-Fe phase significantly affects the structure and evolution of the corrosion interface [13,14]. Wang et al. focused on the corrosion behavior of Fe-B steel in molten zinc at different temperatures, demonstrating that increased temperature weakens the stability of the corrosion product layer, making it more prone to spallation, which exposes fresh metal surfaces and further accelerates the corrosion process [15]. Meanwhile, Kocaman and Ouyang et al. studied the effect of molybdenum on the corrosion resistance of the alloys. Their results showed that the addition of Mo significantly influences the microstructure and phase composition of the coatings, thereby improving their mechanical properties and corrosion resistance [16,17]. Zheng et al. investigated the effect of Fe_2_B orientation on erosion–wear behavior, finding that Fe_2_B phases oriented perpendicularly can effectively resist abrasive cutting, form a stable interface structure, and enhance the alloy’s wear resistance in high-temperature liquid zinc [18]. The iron boride compounds in these alloys neither wet nor react with liquid zinc, ensuring their corrosion resistance. However, these boride phases tend to be inherently brittle, which can limit the overall mechanical performance of the alloys. To address this issue, relevant studies have found that by incorporating elements such as Cr and Al [19,20,21], the brittleness of Fe-B alloys can be mitigated to some extent while significantly improving their high-temperature corrosion resistance. However, despite these advantages, the α-Fe phase, as the matrix phase, still exhibits relatively poor corrosion resistance, which limits the overall corrosion resistance of the alloy in molten zinc. The present study aims to mitigate the detrimental influence of the α-Fe phase on the overall corrosion resistance of the alloy in molten zinc.

Surface aluminizing treatment has gained significant research interest in recent years as a method to improve the corrosion resistance of materials [22,23,24]. By applying aluminizing treatment to Fe-B-Cr-Al alloys, the α-Fe solid solution on the surface can be transformed into iron–aluminum compounds, which are expected to significantly improve corrosion resistance.

This study combines surface treatment with bulk corrosion-resistant materials by applying an aluminizing treatment to the Fe-20Cr-5B-3Al alloy [25,26], a bulk corrosion-resistant material for molten zinc environments. The composition of the aluminized layer was characterized, and the corrosion resistance of the treated alloy was evaluated through a 128 h liquid zinc corrosion test, comparing it with the untreated alloy. This study aims to explore the effect of surface aluminizing treatment on the corrosion resistance of Fe-20Cr-5B-3Al alloy in liquid zinc.

## 2. Materials and Methods

### 2.1. Materials

**Figure 1:**
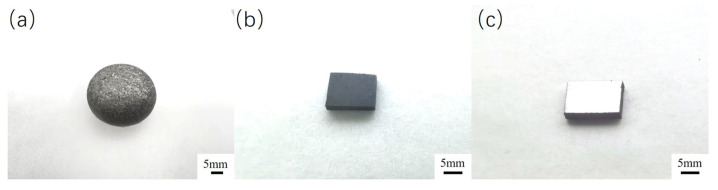
(**a**) As-cast alloy; (**b**) wire-cut alloy; (**c**) polished alloy.

The Fe-20Cr-5B-3Al alloy (Cr: 20 wt.%, B: 5 wt.%, Al: 3 wt.%, Fe: Bal.) was melted in a WF-I-type non-consumable vacuum arc furnace (Henan NBD Technology Co., Ltd., Zhengzhou, China) at a working temperature of approximately 2000 °C. Due to the high tendency of boron to be lost during high-temperature melting, a ferroboron master alloy containing 12 wt.% boron was used as the boron source. To reduce oxygen content, titanium granules were added for deoxidation treatment. In each operation, up to four samples could be melted simultaneously. Titanium granules were first melted for about 4 min to absorb oxygen, followed by the melting of the alloy samples. Each sample was melted for 4 min, repeated twice, then turned over and remelted three more times using a manipulating rod in coordination with a tungsten electrode rod to ensure homogeneity of the alloy composition. A total of eight complete samples were synthesized in this study. Each sample was sectioned from the central part of the alloy and cut into four pieces with dimensions of 14 mm × 7 mm × 2 mm using a DK-7720 electric spark wire cutting machine (Baomade Electromechanical Co., Ltd., Suzhou, China), yielding 32 samples for subsequent experiments. Subsequently, during the polishing process, mechanical grinding was sequentially performed on a PG-2B metallographic pre-grinding machine (Cankang Optical Instrument Co., Ltd., Shanghai, China) using SiC sandpapers with grit sizes of 150, 400, 800, 1000, and 2000. Final polishing was carried out on an LC-250 metallographic polishing machine (Teshi Test Equipment Co., Ltd., Suzhou, China). The polished samples were then ultrasonically cleaned in a JPS-80A cleaner (Youmai Technology Co., Ltd., Shenzhen, China) for two hours to remove surface oils and the surface oxide layer, thereby ensuring the accuracy and consistency of the experimental tests. The samples after melting, cutting, and polishing are shown in Figure 1.

### 2.2. Aluminizing Treatment

The aluminized layer was prepared using the powder pack cementation method. The pack mixture consisted of Al (aluminum powder), Al_2_O_3_ (alumina powder), and NH_4_Cl (ammonium chloride). During the reaction, NH_4_Cl acted as an activator, increasing the concentration of active Al atoms, which enhanced the thickness of the aluminized layer and improved the overall aluminizing effect.

**Figure 2:**
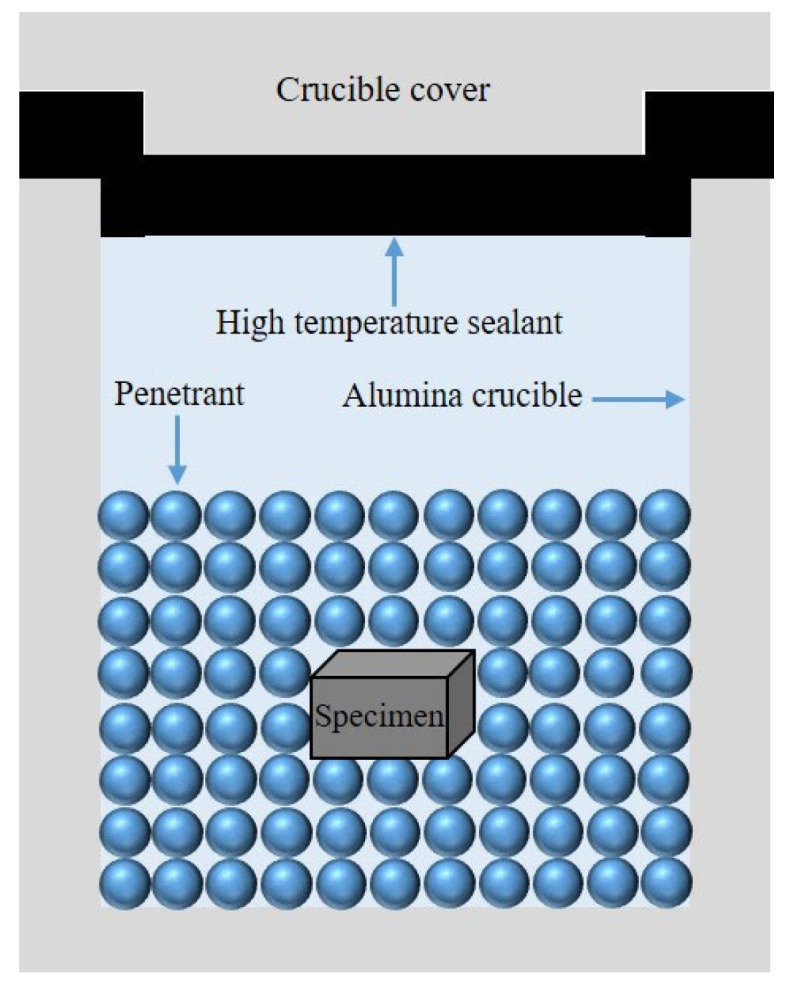
Schematic diagram of the pack cementation aluminizing process.

The specific procedure was as follows: First, the well-mixed pack mixture was placed into an alumina crucible (20 mm × 20 mm × 17 mm), filling half of its volume. The sample was then positioned on top of the pack mixture, ensuring it was centrally placed within the crucible. Subsequently, more pack mixture was added until the sample was completely covered. To enhance sealing, a high-temperature sealing adhesive (HBC-1096) was evenly applied to the lid’s surface. This sealant, produced by Guangzhou Nairui Environmental Protection Technology Co., Ltd. (Guangzhou, China), is resistant to high temperatures and was primarily used to seal the edges of the crucible lids. The crucible was then placed in a drying oven at 100 °C for 1 h to allow the adhesive to fully solidify and to preheat the pack mixture (Figure 2). Finally, the sealed crucible was placed into a box-type resistance furnace for annealing. After the aluminized layer had formed, the sample was allowed to cool to room temperature in the furnace. In this study, we prepared a total of 32 samples, which were divided into two groups: one group consisted of the original untreated samples, and the other group comprised samples treated with aluminizing.

### 2.3. Corrosion Test

**Figure 3:**
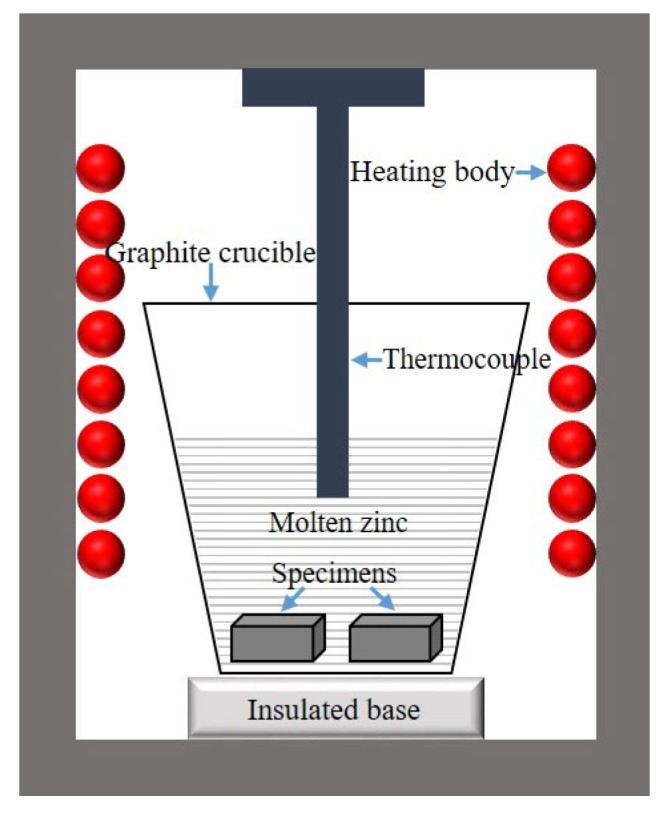
Schematic diagram of corrosion test equipment.

During the zinc corrosion experiment, high-purity zinc ingots (99.98%) were selected and placed in a graphite crucible, which was then placed in a resistance furnace and heated until the molten zinc temperature was precisely maintained at 600 °C (Figure 2). Both the surface-treated alloy and the untreated alloy were simultaneously immersed in the crucible containing molten zinc, ensuring that both alloys were exposed to the same corrosion environment and maintaining consistency in the experimental conditions. After being corroded for different amounts of time, both were taken out at the same time to observe the relevant corrosion conditions (Figure 3). Regarding the calculation of the corrosion layer thickness, specifically, the average corrosion layer thickness (M) was determined by measuring five randomly selected positions within the layer, using the following equation:

(1) $$M=\frac{Z_{1}+Z_{2}+Z_{3}+Z_{4}+Z_{5}}{5}$$

where Z_1_–Z_5_ represent the measured thicknesses at five different random positions within the corrosion layer.

### 2.4. Characterization

Before SEM observation, the samples were first hot-mounted using a BQ-2 metallographic mounting press (Shangcai Testing Machine Co., Ltd., Shanghai, China) at 120 °C for 30 min. The mounted samples then underwent surface preparation, which involved sequential grinding with 150, 400, 800, 1000, and 2000 grit SiC sandpapers on a PG-2B metallographic pre-grinding machine (Shangcai Testing Machine Co., Ltd., Shanghai, China), followed by final polishing using an LC-250 metallographic polishing machine (Trojan Material Technology Co., Ltd., Suzhou, China). The microstructure, composition, and elemental distribution of the alloy, aluminized layer, and corrosion layer were characterized by scanning electron microscopy (SEM) and energy-dispersive spectroscopy (EDS) using a JSM-6510 scanning electron microscope manufactured by JEOL Ltd. (Tokyo, Japan). The probe diameter of the scanning electron microscope was 1 mm and the acceleration voltage was 20 kV. The phase structure was further determined by X-ray diffraction (XRD) analysis, which was carried out using a D/MAX 2500 PCX diffractometer from Rigaku Corporation (Tokyo, Japan). The instrument employed Cu-Kα radiation at 40 kV and 40 mA, with a scanning range of 10–90° and a step size of 0.02°. The XRD data were processed using JADE^®^ 6 software provided by Materials Data, Inc. (Pleasanton, CA, USA), and the charts were plotted using Origin^®^ 2022 by OriginLab (Northampton, MA, USA).

## 3. Results and Discussion

### 3.1. Microstructure of Fe-20Cr-5B-3Al Alloy

**Figure 4:**
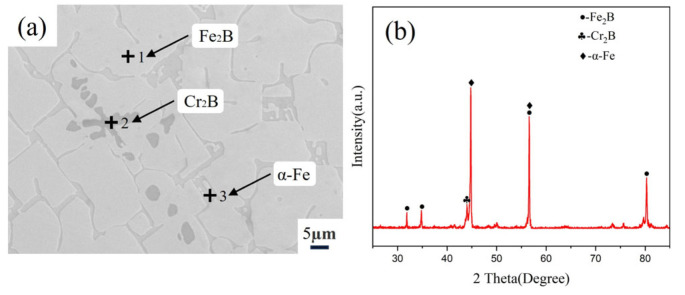
(**a**) Microstructure of the Fe-20Cr-5B-3Al alloy, (**b**) and its XRD pattern.

Through microstructural observation and XRD analysis (Figure 4) of the Fe-20Cr-5B-3Al alloy, it was found that the alloy primarily consists of a large gray-white phase, strip-shaped dark-gray phase, and a small amount of granular black phase. The XRD spectrum shows that the alloy mainly contains Fe_2_B, α-Fe, and Cr_2_B phases. Among these phases, the diffraction peaks of the Fe_2_B and α-Fe phases are relatively strong, indicating that they are present in relatively high proportions in the alloy. In contrast, the diffraction peaks of the Cr_2_B phase are weaker, suggesting a lower content.

**Figure 5:**
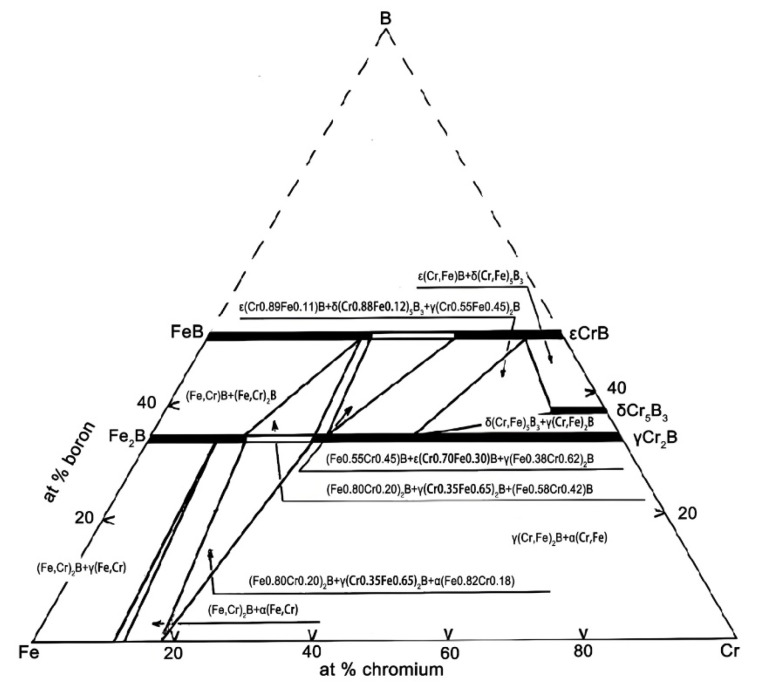
Isothermal section of Fe-Cr-B system at 1373 K [27].

Combining the results from the EDS analysis (Table 1), it can be confirmed that the large gray-white phase corresponds to the Fe_2_B phase, the strip-shaped dark-gray phase corresponds to the α-Fe phase, and the small granular black phase corresponds to the Cr_2_B phase. This represents a general pattern observed on the surfaces of the original sample group analyzed in this study. The EDS results reveal significant differences in elemental composition across the three analyzed regions. Point 1 (Fe_2_B phase) contains approximately 29.65 at.% Cr and 69.08 at.% Fe, indicating a notable dissolution of Cr in the Fe_2_B lattice. Similarly, Point 2 (Cr_2_B phase) is rich in Cr (60.36 at.%) but also contains a substantial amount of Fe (39.48 at.%). Point 3, corresponding to the α-Fe phase, shows a markedly different composition, with 12.91 at.% Al and only 2.05 at.% Cr, while Fe remains dominant (85.04 at.%). This implies that aluminum is preferentially dissolved in the α-Fe matrix. Due to the fact that Al primarily dissolves into the α-Fe matrix phase and does not participate in compound formation, the remaining Fe, Cr, and B elements constitute a ternary system in which a significant number of Cr and Fe atoms can be substitutes for each other in the Fe_2_B and Cr_2_B phases. This behavior is clearly reflected in the Fe-Cr-B ternary phase diagram (Figure 5) [27].

### 3.2. Microstructure of Fe-20Cr-5B-3Al Alloy Aluminizing

**Figure 6:**
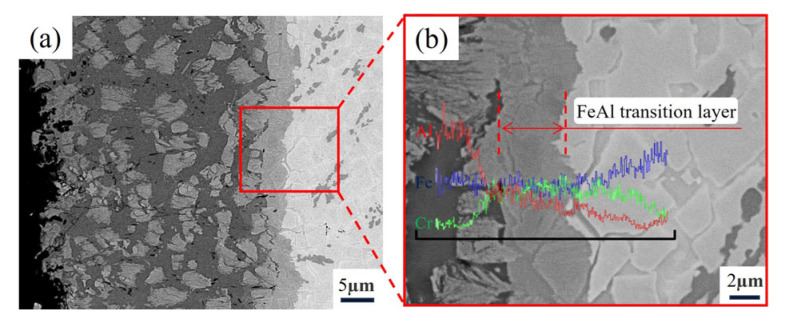
(**a**) Microstructure of the aluminizing layer of the Fe-20Cr-5B-3Al alloy, (**b**) and its EDS line scan.

After aluminizing treatment, a distinct aluminide diffusion layer formed on the sample surface, **as shown in** Figure 6a, **with** a transition zone of certain thickness between the diffusion layer and the substrate. To further clarify the distribution of elements in different regions, an EDS line scan was conducted on the magnified region, **as presented in** Figure 6b. The results reveal that, along the thickness of the diffusion layer, there are generally three distinct regions: the surface-rich Al phase region, the intermediate FeAl transition phase region, and the substrate phase region. This indicates that the formation of the aluminized layer is primarily reflected in the surface-rich Al phase and the intermediate transition zone, with the specific formation of new phases analyzed in more detail through subsequent EDS and XRD analysis.

**Figure 7:**
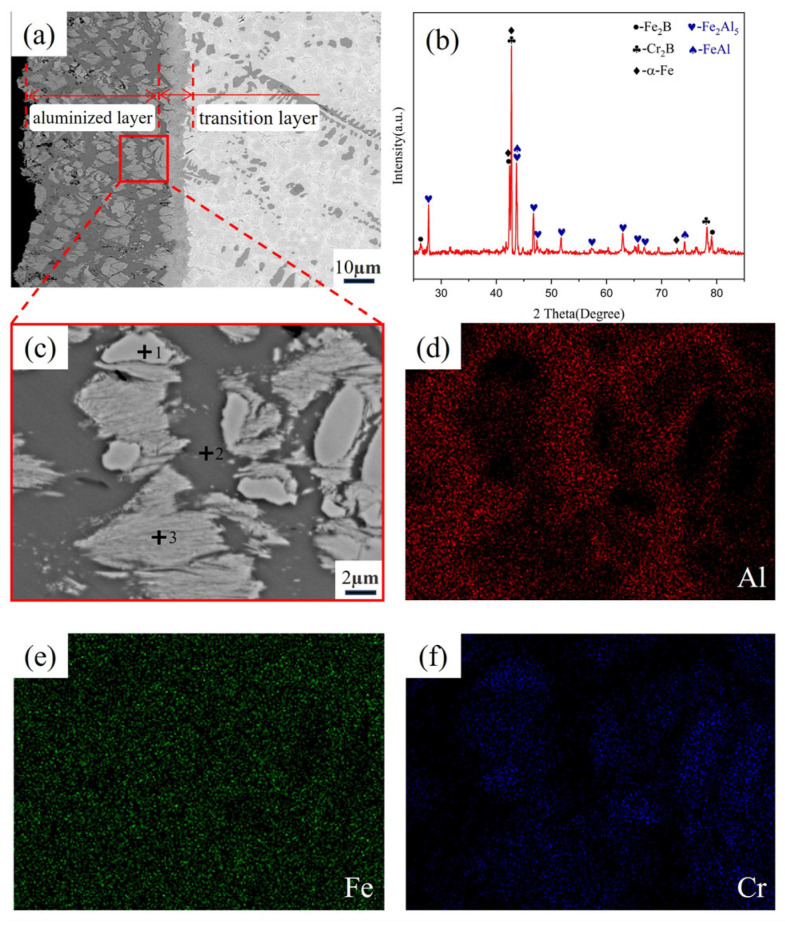
Fe-20Cr-5B-3Al alloy aluminized layer. (**a**) Microstructure of the aluminized layer; (**b**) XRD pattern; (**c**) local magnification of the aluminized layer; (**d**) distribution of Al element; (**e**) distribution of Fe element; (**f**) distribution of Cr element.

As shown in Figure 7, energy-dispersive surface scanning (EDS) and X-ray diffraction (XRD) analysis of the Fe-20Cr-5B-3Al alloy revealed the complex microstructure of the aluminized layer and its interactions. According to the results of the EDS quantitative analysis (Table 2), the main phases in the aluminized layer are Fe_2_Al_5_ and the transition phase FeAl. The XRD pattern shows multiple strong diffraction peaks corresponding to Fe_2_Al_5_, indicating its dominance in the aluminized layer, whereas the diffraction peaks of FeAl are fewer and noticeably weaker in intensity. The specific element distribution is as follows:

At Point 1, the primary elements are Al (0.33% at.%), Cr (61.98% at.%), and Fe (37.69% at.%). A comparison of the surface scan images Figure 7d,f shows that in the Cr-enriched region, the concentration of Al is low. Based on the analysis of the matrix composition, this phase corresponds to the Cr_2_B phase in the matrix.

At Point 2, a clear aluminum-rich feature is observed, with Al content reaching 69.15 at%, Cr at 0.89 at.%, and Fe at 29.96 at.%. The main phase here is Fe_2_Al_5_. This result indicates that the rapid diffusion of aluminum has caused the transformation of the original α-Fe phase in the matrix into Fe_2_Al_5_.

At Point 3, the elemental analysis shows Al at 35.28 at.%, Cr at 12.55 at.%, and Fe at 52.17 at.%. This region shows an elongated gray phase and black phase and is identified as a mixture of Fe_2_Al_5_ and Fe_2_B phases. This conclusion is supported by several factors. First, magnified observations of the aluminized layer show that during aluminum penetration, the Fe_2_B phase undergoes fragmentation, forming many small fragments. Additionally, although the EDS point analysis is conducted at a specific point, it actually represents a small area. The aluminum content in this region is lower than that of Fe_2_Al_5_, but it is clearly higher than that of Fe_2_B, which can be attributed to the region containing both Fe_2_Al_5_ and fragmented Fe_2_B phases. Finally, XRD analysis did not reveal the discovery of any new ternary compound between Fe-B-Al, further supporting the coexistence of Fe_2_Al_5_ and Fe_2_B phases.

In conclusion, the penetration of aluminum not only facilitated the formation of the Fe_2_Al_5_ phase but also significantly influenced the microstructure of the matrix phase. The transformation of the α-Fe phase into Fe_2_Al_5_ destroyed the integrity of the Fe_2_B phase, while the Cr_2_B phase structure remained intact, indicating the superior stability of chromium during the high-temperature aluminizing process.

### 3.3. Microstructure of the Alloy After Zinc Liquid Corrosion

**Figure 8:**
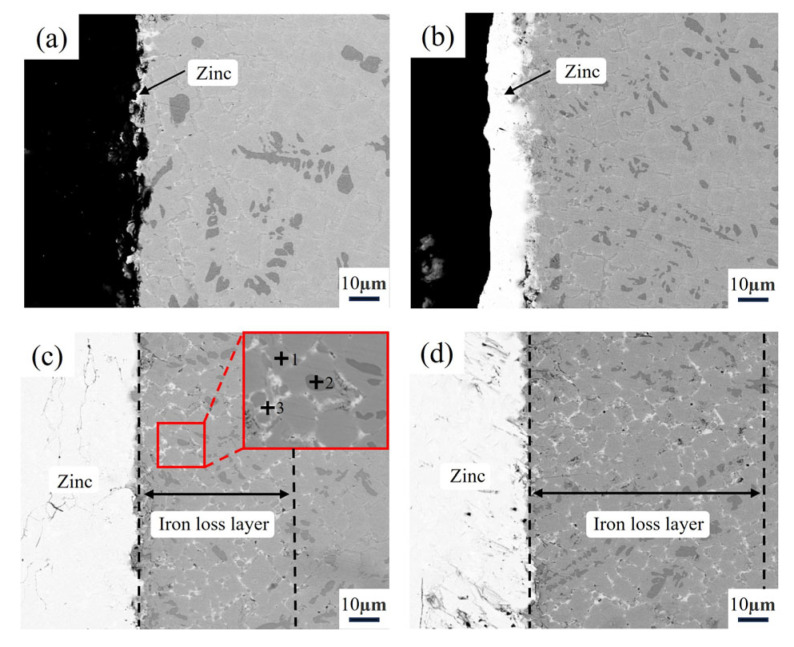
Cross-sectional morphology of Fe-20Cr-5B-3Al alloy after immersing in zinc liquid at 600 °C for different time: (**a**) 16 h, (**b**) 32 h, (**c**) 64 h, (**d**) 128 h.

According to Figure 8a, during the 16 h corrosion test, only a small amount of zinc adhered to the surface of the sample, with the surface remaining mostly non-wetted by the molten zinc. The matrix was not significantly affected by corrosion from the zinc liquid.

When the test duration was extended to 32 h, as shown in Figure 8b, a thin layer of zinc deposition began to appear on the sample surface, disrupting the non-wettability between the sample and molten zinc. Additionally, a slight corrosion phenomenon was observed at the interface between the matrix and the zinc liquid.

After 64 h of testing, as shown in Figure 8c, a noticeable corrosion layer had formed on the sample surface. Local magnification observation via electron microscope and EDS analysis results from Table 3 revealed that the original Fe_2_B and Cr_2_B phases in the matrix remained intact. In Point 1 (Fe_2_B phase), the EDS data show 74.28 at.% Fe and 24.14 at.% Cr, with a small amount of Zn (0.23 at.%), indicating that the Fe_2_B phase is resistant to molten zinc. In Point 2 (Cr_2_B phase), the Zn content was even lower (0.12 at.%), suggesting that this phase also possesses excellent corrosion resistance against molten zinc. However, the α-Fe phase exhibited poor corrosion resistance and was easily corroded by the molten zinc. At Point 3, the Fe content was 8.63 at.%, and the Zn content was 85.14 at.%, with a ratio of approximately 1:10. The corrosion product formed by the reaction between α-Fe and molten zinc was FeZn_10_. Ultimately, the corrosion spread along the α-Fe phase in a tentacle-like pattern, by passing the Fe_2_B phase and diffusing into the matrix, forming a corrosion layer of approximately 61.2 μm.

After 128 h of corrosion, as shown in Figure 8d, the thickness of the corrosion layer continued to increase, reaching approximately 112.6 μm. Based on the above results, it can be concluded that the corrosion of the sample by molten zinc primarily occurs along the α-Fe phase, and as time progresses, the degree of corrosion deepens.

### 3.4. Microstructure of the Aluminizing Alloy After Zinc Liquid Corrosion

**Figure 9:**
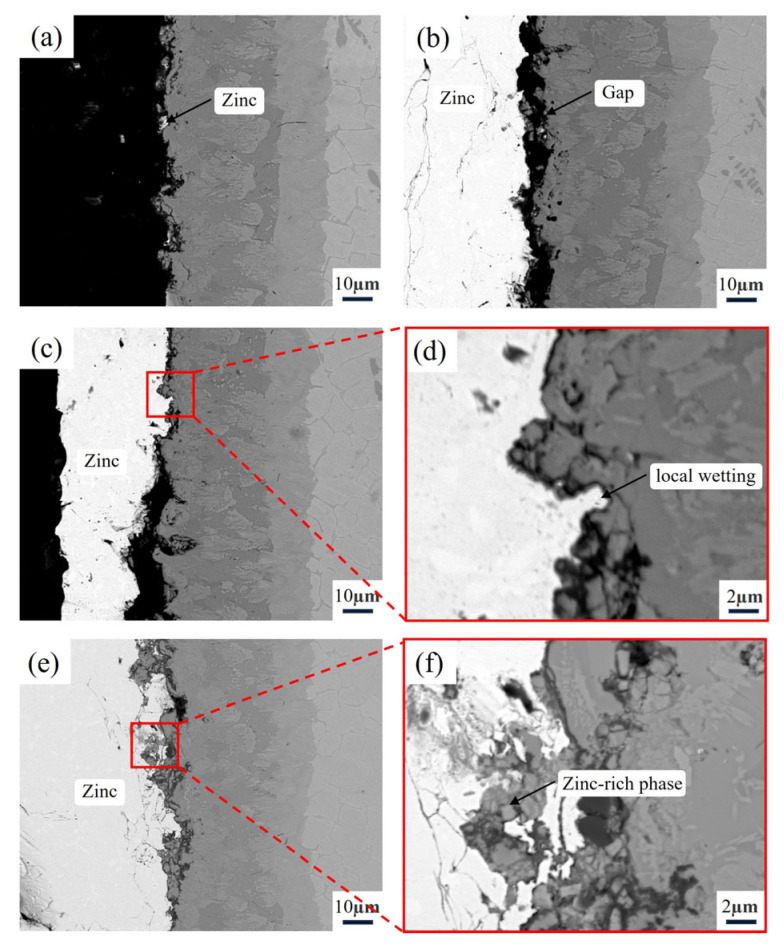
Cross-sectional morphology of the aluminized alloy after immersing in zinc liquid at 600 °C for different time: (**a**) 16 h, (**b**) 32 h, (**c**) 64 h, (**d**) 64 h local magnification, (**e**) 128 h, (**f**) 128 h local magnification.

According to Figure 9a, after 16 h of contact with molten zinc, the surface of the sample treated with aluminizing showed no significant zinc adhesion, demonstrating good non-wettability. When the corrosion time was extended to 32 h, as shown in Figure 9b, a zinc layer began to form on the sample surface. However, electron microscope observations revealed that there was still a noticeable gap between the zinc layer and the aluminized layer, indicating that the molten zinc did not establish substantial contact with the aluminized layer. Despite the zinc layer gradually increasing in thickness, the sample still did not exhibit wettability with the molten zinc, suggesting that, in the early stage of corrosion, the aluminized layer maintained non-wettability with the molten zinc.

After 64 h of corrosion, as shown in Figure 9c,d, although in some localized areas, complete wetting of the aluminized layer by molten zinc was observed, no significant corrosion occurred, and the integrity of the aluminized layer was preserved. When the corrosion time reached 128 h, as shown in Figure 9e, the zinc layer thickness had significantly increased, and the molten zinc had fully wetted the aluminized layer. From the localized magnification in Figure 9f, it can be observed that the molten zinc had started to corrode the aluminized layer. Some grain boundaries near the molten zinc in the aluminized layer underwent separation due to the formation of zinc-rich phases (Zn 34.48 at.%) at local grain boundaries, causing partial fracture of the aluminized layer, which then entered the molten zinc. The aluminized layer exhibited a thickness loss of approximately 23.9 μm. However, the matrix was not affected, primarily because, on one hand, the surface aluminizing treatment transformed the α-Fe phase of the matrix into the more corrosion-resistant Fe_2_Al_5_ phase. On the other hand, the Fe_2_Al_5_ phase was distributed with a large amount of Cr_2_B phase and fragmented fine Fe_2_B phases, which further enhanced the corrosion resistance of the aluminized layer, effectively preventing corrosion of the matrix by molten zinc along the α-Fe phase.

### 3.5. Zinc Liquid Corrosion Mechanism

**Figure 10:**
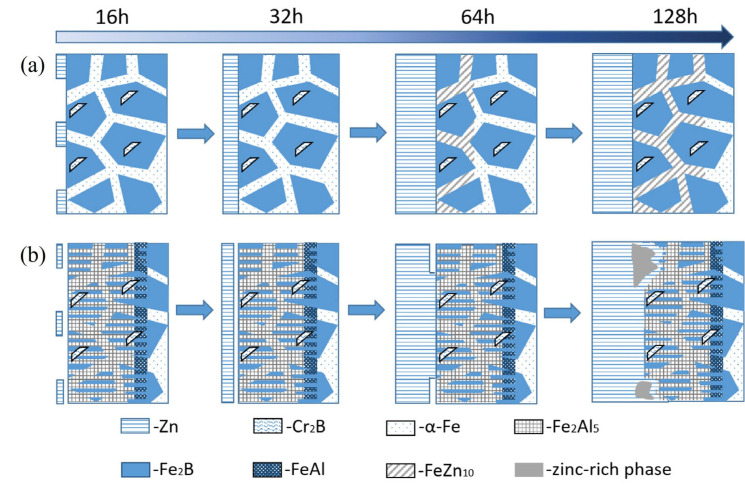
Zinc liquid corrosion mechanism: (**a**) Fe-20Cr-5B-3Al alloy, (**b**) aluminized Fe-20Cr-5B-3Al alloy.

In the zinc solution, the corrosion process of the original alloy (a) and the aluminized alloy (b) is shown in Figure 10. The corrosion process of the original alloy can be divided into four stages: First, the wetting process between the molten zinc and the alloy surface begins. Although most of the alloy surface remains unwetted, a small amount of zinc adheres to the surface. Over time, a thin zinc layer gradually forms on the alloy surface. As time progresses, the thickness of the zinc layer increases, and the molten zinc reacts with the α-Fe phase to form FeZn_10_. This reaction extends along the α-Fe phase into the matrix, forming a corrosion layer. Finally, as the corrosion time increases, the thickness of the corrosion layer also gradually increases.

After surface aluminizing treatment, the Fe-20Cr-5B-3Al alloy exhibits significantly improved zinc corrosion resistance compared to the original alloy. The corrosion process of the aluminized alloy can also be divided into four stages: First, there is the wetting process of the molten zinc. However, unlike the untreated original alloy, this process takes longer. At around 64 h, only parts of the surface are wetted, and it is not until 128 h that the surface is fully wetted. At this point, the aluminized layer begins to be affected by the molten zinc corrosion. This corrosion mainly occurs in the form of localized zinc-rich areas on the surface of the aluminized layer breaking off, with no iron loss layer observed, and the matrix remains unaffected. Similar results were reported by Yan et al. [28], who studied the corrosion behavior of plasma-sprayed Fe_2_Al_5_ coatings in molten zinc and found that the primary failure mechanism was the formation of zinc-rich liquid phases at local grain boundaries, which led to partial dissociation and spalling of the coating into the molten zinc.

## 4. Conclusions

This study first analyzed the microstructure of the Fe-5B-20Cr-3Al alloy and surface aluminized the alloy using the powder pack cementation method. The microstructure of the aluminized layer was then examined. Afterward, both the original and aluminized alloys underwent a 128 h zinc liquid corrosion test. Based on the research results, the following conclusions can be drawn:

(1) The homogenized Fe-20Cr-5B-3Al alloy consists of large grayish-white Fe_2_B, strip-like dark-gray Cr_2_B, and a small amount of granular α-Fe. The solubility of Cr and Fe in Fe_2_B and Cr_2_B is relatively high. The α-Fe solid solution contains a significant amount of aluminum and a small amount of chromium, indicating that Al mainly dissolves into the α-Fe matrix phase. After the alloy was corroded in zinc liquid at 600 °C for 128 h, the α-Fe phase, which has poor corrosion resistance, easily reacted with the zinc liquid to form the corrosion product FeZn_10_. The zinc liquid bypassed the Fe_2_B phase and continued to corrode the matrix along the α-Fe phase, severely affecting the alloy’s resistance to zinc liquid corrosion.

(2) After surface aluminizing treatment, a dense and continuous aluminized layer formed on the Fe-20Cr-5B-3Al alloy surface. The α-Fe phase with poor corrosion resistance was transformed into the more corrosion-resistant Fe_2_Al_5_ phase. The original Fe_2_B phase broke into finer structures due to the aluminizing treatment and dispersed over the Fe_2_Al_5_ phase. Cr_2_B remained unaffected and maintained its structural integrity. This transformation significantly improved the alloy’s resistance to zinc liquid corrosion, protecting the α-Fe phase in the matrix from corrosion.

(3) Furthermore, the untreated alloy samples quickly wetted in the zinc liquid, while the aluminized samples took a longer time to become completely wetted. This delay is not only related to the surface morphology of the aluminized samples but may also be attributed to the surface effects of the liquid zinc.

## Figures and Tables

**Table 1:** EDS analysis results of the alloy (at.%).

Elements	Al	Cr	Fe	Phase
Point 1	1.27	29.65	69.08	Fe_2_B
Point 2	0.16	60.36	39.48	Cr_2_B
Point 3	12.91	2.05	85.04	α-Fe

**Table 2:** EDS analysis results of the alloy after aluminizing treatment (at%).

Elements	Al	Cr	Fe
Point 1	0.33	61.98	37.69
Point 2	69.15	0.89	29.96
Point 3	35.28	12.55	52.17

**Table 3:** EDS analysis results of the alloy after liquid zinc corrosion (at%).

Elements	Al	Cr	Fe	Zn
Point 1	1.35	24.14	74.28	0.23
Point 2	0.14	61.38	38.36	0.12
Point 3	1.97	4.12	8.63	85.28

## Data Availability

The original contributions presented in this study are included in the article. Further inquiries can be directed to the corresponding author.
